# Narrative Matters: Cultural humility in mental healthcare

**DOI:** 10.1111/camh.70059

**Published:** 2025-12-23

**Authors:** Ade Kolade

**Affiliations:** ^1^ University of Manchester Manchester UK

**Keywords:** Child and adolescent mental health service, Cultural Humility, Cultural Competence, Ethnic Minority Young People

## Abstract

Young people from ethnic minority backgrounds in the United Kingdom are less likely to seek psychological support than their white peers. When they do engage with services, a disproportionate number leave, often prematurely. Intercultural frameworks have been developed to help clinicians engage sensitively with diverse populations and improve outcomes. For decades, ‘cultural competence’ has been an accepted intercultural framework across the United Kingdom and beyond (North America, Europe and Australia). However, despite its wide application, ethnic disparities in mental healthcare remain virtually unchanged, exposing a gap between the promise of cultural competence and what it delivers. This article endorses ‘cultural humility’: a different approach to intercultural practice, inviting a ‘way of being’ embedded into practice rather than an acquired skill set. It will outline theoretical principles and describe how this model can be applied to clinical settings, using real‐life scenarios involving ethnic minority young people.

## Introduction

Ethnic minority youth remain under‐represented in the UK mental health system, despite being disproportionately exposed to a cluster of adverse experiences (e.g. social deprivation, traumatisation, racism). Clinical sensitivity to the factors that influence the way ethnic minority youth engage with mental health services remains a public health concern (Nwokoroku, Neil, Dlamini, & Osuchukwu, 2022). ‘Cultural competence’ has been taught for over three decades (Davis et al., 2018) and is a commonly used framework for intercultural practice. It entails training professionals to engage sensitively with patients from diverse populations and improving awareness of different norms and values in ethnic minority communities.

However, raising cultural awareness alone may be insufficient because, despite evidence of good will and investment, mental health outcomes for ethnic minority young people remain virtually unchanged, with a majority still reporting poorer outcomes compared to their white British peers (Kapadia et al., 2022), with:

1. Lower rates of engagement in clinical services;
2. Higher rates of premature drop‐out from clinical intervention;
3. Lower rates of responsiveness to the full range of clinical treatments;
4. Higher rates of dissatisfaction with clinical treatment (Beck, Naz, Brooks, & Jankowska, 2019).

These outcomes demonstrate an urgent need to do things differently, as cultural competence – either as a conceptual framework or training package – has done little to rectify ethnic disparities that have long beset UK mental healthcare.

## Cultural humility: A call to do things differently

Cultural humility moves away from the idea that one is ever ‘culturally competent’; competence suggests a false endpoint in therapists' learning. This paper argues that there is a fluid and complex relationship between culture and mental health (Davis et al., 2018). Culture is neither a fixed entity nor a phenomenon with immutable laws that can be learnt and applied in clinical practice. Rather, culture is a dynamic process, changeable over space and time and relative to the individual. The relationship between culture and mental health is often highly individual: some see their cultural background as a protective factor (e.g. a source of pride and belonging), while others perceive it as a risk (e.g. discrimination experiences) (Beck et al., 2019). The only constant in culture is change, especially in the context of mental health.

Cultural humility considers intercultural practice a process. It encourages clinicians to feel less apprehensive about culturally unfamiliar terrain by removing the expectation that they must appear well informed in areas of cultural importance to ethnic minority young people (Davis et al., 2018). The framework, therefore, encourages honesty about the limits of knowledge. It requires therapists to demonstrate a willingness to learn from culturally diverse patients, which requires both *intra‐* and *inter‐*personal work (Davis et al., 2018). Intra‐personal work is underpinned by a continuous process of self‐reflection on one's own cultural background, identities, biases, assumptions and attitudes when working with culturally diverse patients. Inter‐personal work entails a reflexive openness to learning about other cultures. In addition to engaging with the academic literature, it will include direct learning from culturally diverse patients. Therapists must be encouraged to be *professionally curious* about the relationship between culture and mental health; raising the issue even if the patient does not raise it themselves. Avoiding these conversations may be more detrimental to the therapeutic relationship rather than the reportedly small risks of having them (Beck et al., 2019). It is important to consider *how* these conversations are initiated and managed by the therapist. They require sensitivity, confidence and, perhaps most importantly, relinquishing ‘the expert role’ on cultural experience. The ‘insider epistemology’ is a position that only patients truly access and articulate. Cultural sensitivity, therefore, is not a pre‐existing therapist quality, but rather a ‘way of being’ involving openness, humbleness and a willingness to learn from culturally diverse patients (Orlowski, Moeyaert, Monley, & Redden, 2025).

Cultural humility embraces complexity and resists generalisation. It requires an understanding of the variations in cultural experience, mental health experience and different degrees of engagement with services (Nwokoroku et al., 2022). Without this understanding, prejudices about cultural experience are likely to affect clinical practice, to the detriment of patients. Healthcare is far from impervious to the propagation of stereotypes about ethnic groups, evidenced by the long and distressing history of medical racism. Clinicians may make assumptions about their clients' experiences based on positive or negative stereotypes, believing their practice to show cultural competence but it only serves to violate the individuality of the patient (Tervalon & Murray‐Garcia, 1998). Consider, for example, reports of unconscious bias in obstetric care leading to disproportionate black maternal mortality rates (Odems et al., 2024). Cultural humility places a premium on the individual's experience while acknowledging their complex and sometimes conflicting relationship to a broader culture.

## Cultural humility in practice

Theoretical frameworks may lose integrity in day‐to‐day clinical practice, particularly in the case of psychotherapy. There are well‐established patterns showing a lack of adherence to treatment protocols in a variety of therapeutic settings (Greer, 2002). Real‐world practicalities, including insufficient resources, may impact theory‐driven practice, undermining the quality and continuity of care (Greer, 2002).

Cultural humility can be applied to most therapeutic modalities (Orlowski et al., 2025; Singh et al., 2022) and shares approaches with established models. For example, cultural humility involves both collaborative empiricism and a high degree of person‐centredness in the way it empowers the young person to define their own reality. It can therefore augment the effects of different therapeutic approaches and techniques, but perhaps more indirectly via the strengthening of the therapeutic connection that the clinician has with the young person. Young people may then have greater confidence in the clinician's judgement because complex aspects of their experience were handled with sensitivity and care. This may be in contrast to prior negative experiences where this was either overlooked or dismissed by professionals (Orlowski et al., 2025).

Cultural humility differs from established therapeutic models in the salience of culture in therapeutic conversations and a full acceptance of the reality of societal stressors that affect ethnic minority young people specifically, such as racism. For example, a clinician of any therapeutic background could offer therapeutic support to a Muslim patient and be ignorant of, even sceptical about, the existence of Islamophobia. This clinician, however, may only be able to support the young person in a limited way as their lived experience will not be sufficiently explored or even invalidated by the clinician. Such experiences not only lead to suboptimal therapeutic outcomes (Kapadia et al., 2022) but also dis‐engagement from mental healthcare among diverse young people (Beck et al., 2019).

## Clinical scenarios featuring cultural humility

Here, clinical scenarios illustrate the application of cultural humility in child and adolescent mental health services (CAMHS) in the United Kingdom.

*Scenario 1*: An Urdu‐speaking parent challenges a therapist on the conventional understanding of mental illness in Western medicine, and their beliefs about the ways in which it manifests in the mind, body and soul. The parent is so disturbed by the differences in perspectives that they refuse to consent to their child receiving much‐needed psychological care.

The cultural humility position implies there are limits to clinical expertise in psychotherapeutic practice and healthcare. If there is patient–therapist consensus on the approach (e.g. CBT) and the therapeutic outcome, there should be flexibility in understanding how the patient became unwell, using a framework which can accommodate their religious and spiritual beliefs and a communication style that is culturally sensitive, collaborative and unfixed.

This approach recognises that current theories may be insufficient as they draw from incomplete or contradictory evidence. We should work within the limits of what is established in the existing evidence base (such as the link between behaviour and mood), while considering other cultural explanations for the onset of mental health problems. The subjective definition of mental ill health will likely contain different religious and spiritual interpretations in diverse and ethnic minority groups. This is a crucial framework when engaging with diverse populations, which will diminish feelings of cultural superiority that may, unconsciously or otherwise, influence communication in multicultural settings.

It is important to ‘treat the patient, not the illness’, because one cannot treat the illness without engaging with the patient first. The low rates of engagement among ethnic minority young people underscore this point. Sensitivity to individual values or/and cultural norms is vital in engagement and adherence processes. It requires a young person to have trust and confidence in a clinician's ability, which is likely lacking without cultural humility. Demonstrating qualities that promote dialogue, collaboration and mutual respect (such as active listening or openness) when engaging with diverse patients and their families is key.

*Scenario 2*: A South Asian young person feels that they have lost connection to their faith, which they have kept private because they think this will disappoint their family. The issue particularly impacts their quality of life, as the young person often feels pressured to spend more time practising religious duties than they do enjoying teenage life with their friends.

Cultural humility implies flexible approaches to patient dilemmas. It is distinct from other intercultural practice frameworks because it includes the idea that culture is fluid, changeable and specific to the individual. Practice cannot be fixed when culture remains unfixed. In Scenario 2, there is no predetermined answer to this culture‐specific question. Indeed, trying to persuade this young person's family about the benefits of non‐religious activities may be counterproductive and the participating family could withdraw from therapy. However, avoiding this sensitive issue may lead to other adverse outcomes, such as poor engagement due to a young person's belief that therapy is fundamentally ineffective.

To maximise clinical outcomes in this scenario, a collaborative exploration of the cultural factors operating on an individual, familial and community level needs to be conducted with the young person to promote engagement. The therapist should aim to work with cultural issues, rather than against them to produce the best possible outcome for the young person given their circumstances. The therapist might discuss the possibility of including some religious activities in a young person's routine – not simply to placate their family but because it might also help them reconnect with their family and community. Therapy might involve reviewing the importance of developmentally appropriate activities with the family (such as watching sports together) despite these activities being religiously unimportant but not altogether prohibited. Discussion might explore if these activities should be prioritised over religious practices, but this will depend on the attitudes of a family, requiring culturally sensitive dialogue in each case. Finally, consider consulting with leaders from the young person's community (while retaining confidentiality) to explore ways in which a sensitive issue could be managed in a culture‐specific way that may be unknown to the therapist. This might include behaviours that traditionally show respect, meeting in venues that are comforting to families, adapting language to promote therapist‐family harmony. The young person should be central in any decision about the handling of cultural issues, as the object of cultural humility is not to substitute the community for the individual, but to recognise and respect individuality in the context of community.

## Conclusion

Since the therapeutic relationship is the most powerful predictor of positive therapeutic outcomes, cultural humility may be an important ‘active ingredient’ for successful recovery among young people from diverse cultural backgrounds. Individual factors which shape a young person's background, identity and values require nuanced delivery of therapeutic interventions. Cultural humility goes beyond mere ‘good clinical practice’, as it requires a shift in power and expertise away from the clinician and towards the young person – a concept perhaps uncomfortable to those trained to feel ‘competent’.

## Conflict of interest statement

The author has no conflicts of interest to disclose.

## Funding information

The author declares no funding.

## Ethics statement

No ethics approval was required for this debate article.

## Data Availability

Data sharing is not applicable to this debate article as no data was created or analysed.
